# DNA methylation-estimated phenotypes, telomere length, aging and risk of intracranial aneurysms: Evidence from genetic studies

**DOI:** 10.1016/j.ibneur.2026.05.009

**Published:** 2026-05-30

**Authors:** Xin Zhang, Aierpati Maimaiti, Meixia Xie, Mirzat Turhon, Naibijiang Yalikun, Wenbao Liang

**Affiliations:** a Department of Neurosurgery, The Fourth Affiliated Hospital of Xinjiang Medical University (Xinjiang Uyhgur Autonomous Region Hospital of Traditional Chinese Medicine), Urumqi, Xinjiang, PR China; b Department of Neurosurgery, The First Affiliated Hospital of Xinjiang Medical University, Urumqi, Xinjiang, PR China; c Department of Health Management Center, The Fourth Affiliated Hospital of Xinjiang Medical University (Xinjiang Uyghur Autonomous Region Hospital of Traditional Chinese Medicine), Urumqi, Xinjiang, PR China; d Department of Interventional Neuroradiology, Beijing Neurosurgical Institute, Capital Medical University, Beijing, PR China; e Department of Neurosurgery, Beijing TianTan Hospital, Capital Medical University, Beijing, PR China; f Department of Neurosurgery, Hotan district People’s Hospital, Hotan, Xinjiang, PR China

**Keywords:** Intracranial aneurysms, DNA methylation-estimated phenotypes, Telomere length, Aging, Mendelian Randomization

## Abstract

**Background:**

The risk of intracranial aneurysm (IA) is increased in older population, suggesting a role for aging. To evaluate the association of genetic variants linked to DNA methylation-estimated phenotypes, telomere length, and aging, with the risk of IA by employing two-sample Mendelian randomization.

**Methods:**

Sex-specific summary-level outcome data were extracted from the GWAS of IA, including 23 cohorts with a total of 5140 cases and 71934 controls. All the study participants were of European ancestry. To improve validity, five varying Mendelian randomization techniques were used in the analysis (MR-Egger, weighted median, inverse variance weighted, simple mode, and weighted mode).

**Results:**

There was a suggestive negative causal relationship between Intrinsic epigenetic age acceleration and unruptured IA (P = 0.022, OR=0.906 [95% CI, 0.83–0.99]), though this did not remain significant after multiple testing correction (corrected P threshold=0.00208). No causal effect was observed between any other methylation-estimated phenotype and aSAH or UIA (all P > 0.05 after correction). A suggestive causal relationship between longevity and aSAH (P = 0.020, OR=1.100 [1.01–1.19]) was observed, with a 9.95% increase in disease risk per 1-SD increase in age, but this also did not withstand multiple testing correction. Each 1-SD increase in telomere length was associated with a 0.9% increase in DNA methylation-estimated granulocyte proportions (P = 0.0025, OR=1.009 [1.003–1.015]) and a 55% increase in intrinsic epigenetic age acceleration (P = 0.018, OR=1.552 [1.080–1.551]), with the former remaining significant after correction.

**Conclusion:**

A negative causal relationship between intrinsic epigenetic age acceleration and IA suggests that an increase in intrinsic apparent age acceleration reduces the risk of IA. The underlying mechanisms and their potential to lower the prevalence of IA as an intervention target require further research.

## Introduction

An intracranial aneurysm (IA) is a relatively common life-threatening disease, usually located at a branch of an intracranial artery. It includes ruptured aneurysms and unruptured intracranial aneurysms (UIAs). In addition, 3.2% of the population is affected by this disease (Vlak et al., 2011). Subarachnoid hemorrhage (SAH), a serious type of stroke, results from the rupture of an IA (van Gijn et al., 2007). Even though accounting for 5–10% of all stroke cases in the United States (Rincon et al., 2013), aneurysmal SAH may have a high mortality rate, and survivors may experience chronic neurophysiological events and a decline in quality of life (Taufique et al., 2016, Shi et al., 2020).

A lot of advancements have been made in the field of epigenetics of IA recently. The number of genes and mutations known to cause familial IA was increased by family-based research, and 17 independent and replicated loci across the genome with an influence on IA risk were discovered by GWASs, particularly those carried out in significant collaborative efforts (Bakker et al., 2020). This IA epigenetic research can characterize the biology and etiology of IA and suggest potential targets for treatment. In addition to these genetic studies, higher genetically predicted SHBG (sex hormone-binding globulin), bioavailable testosterone (BioT) (Molenberg et al., 2022), serum calcium (S-Ca), 25-hydroxyvitamin D (S−25OHD) (Zhang et al., 2023) levels can result in increased vulnerability to developing aneurysmal SAH. Moreover, sex (Vyas et al., 2021), smoking, high-fat diet, hypertension (Vlak et al., 2013), tobacco & alcohol consumption (Zeng et al., 2023), and other factors can result in a greater risk of developing IA by influencing the expression of relevant traits. In addition, the risk of IA and relevant hemorrhage is reduced by elevated serum magnesium levels (Larsson and Gill, 2021). However, the relationship of IA in epigenetic regulatory mechanisms is not fully defined.

Epigenetic modifications commonly implicated in the heritable transmission of a phenotype is DNA methylation (Leontis and Westhof, 2014). Without changing the DNA sequence, it may induce a specified, reversible, and heritable gene expression pattern on differentiating cells (Muotri and Gage, 2006). The most common DNA methylation changes are the addition or removal of methyl groups at the cytosine-phosphor-guanine (CpG) site (Jones et al., 2015, Bird, 2007). An earlier study discovered that Horvath outlined 353 methylation regions that might accurately predict a person's age (Abbott, 2018). Several other methylation sites were identified after this discovery, with different accuracy in predicting age, primarily dependent on the number of sites present (Horvath, 2013). It is thought that individual differences between chronological age and DNA methylation age (DNAm age) somehow reflect biological aging (Horvath et al., 2015, Breitling et al., 2016, Horvath and Raj, 2018, Nwanaji-Enwerem et al., 2018). Thus, a different epigenetic clock can serve as a vital complement to already available biological age measurements, including telomere length or biomarker combinations. There still lies uncertainty regarding the mechanisms underlying changes in methylation levels at certain CpG sites. It is unlikely that DNAm age fully reflects the mitotic clock, as its age can be determined even in nonproliferating tissues (Horvath, 2013). Among the varying techniques for determining an organism's age, the recently discovered epigenetic clock seems to be the most favorable and accurate (Hannum et al., 2013, Vidal-Bralo et al., 2016).

DNAm age is linked to a variety of phenotypes and diseases, including blood counts, diminished lung function, cardiovascular disease, cognitive impairment, frailty, self-rated health, loss of balance, motor incoordination, facial aging, Down syndrome, HIV infection, BMI, and other markers for the metabolic syndrome and obesity (Vetter et al., 2019). Blood cell telomere length (TL) has received substantial research as a biomarker of human aging and a possible risk factor for age-associated illnesses (Demanelis et al., 2020). It has been demonstrated that DNAm age and TL independently correlate with morbidity, chronological age, and mortality (Marioni et al., 2018). There is no evidence from studies showing an association between DNA methylation-estimated phenotypes, telomere length, aging, and increased or decreased risk of IA.

Mendelian randomization (MR) is a technique that assesses the causal relationship between exposure and outcome while reducing the influence of cognitive, social, psychological, and other factors. It leverages genetic variety in non-experimental data (Davey Smith and Hemani, 2014). Many MR studies on IAs have recently surfaced to bring forth new clinical evidence (Molenberg et al., 2022, Zhang et al., 2023, Sun et al., 2022, Bakker et al., 2023, Ma et al., 2022, Zhang et al., 2022). This demonstrates that MR is a credible research technique to address several questions. This study used recently published DNA methylation-estimated phenotypes, telomere length, aging summary data, and IA summary data in a genome-wide association study (GWAS) to assess DNA methylation-estimated using two-sample MR phenotypes, telomere length, aging, and IA using two-sample MR.

## Methods

### Study design

The causal relationship between six DNA methylation-estimated phenotypes, telomere length, aging, and IAs was explored using a two-sample MR approach. Since methylation level and telomere length correlate with aging, the relationship between the three was further investigated to find the direct risk factors for IAs (Supplementary Figure 1).

### Genome-wide association study (GWAS) summary statistics data acquisition

The analysis was performed using the GWAS summary data from published studies, which is available on open access in Supplemental Material (Supplemental raw data) of the original studies. All corresponding studies were conducted with necessary ethical permission and full participant consent.

### Exposure data

The GWAS summary data of six DNA methylation-estimated phenotypes (DNA methylation-estimated granulocyte proportions, DNA methylation GrimAge acceleration, DNA methylation Hannum age acceleration, DNA methylation-estimated plasminogen activator inhibitor−1 levels, DNA methylation PhenoAge acceleration and Intrinsic epigenetic age acceleration), telomere length and aging as exposure (Data sources are shown in Table 1) was obtained, and SNPs linked to the exposure at the genome-wide significance level (*P* < 5e−8) were determined. The independence of SNPs was tested using a stringent criterion (r2 = 0.001; clumping window=10 000 kb). Where an instrumental SNP was not present in the outcome data set, it was eliminated or substituted with an appropriate proxy SNP (r2 > 0.8 in the European 1000 Genomes Project reference panel using LDlink [https://ldlink.nci.nih.gov/]). Then, palindromic SNPs with uncertain allele frequencies (0.42–0.58) were eliminated, and SNP alleles were harmonized across studies.

**Table 1:** Data sources of the intracranial aneurysms–related risk factors.

Traits	sample_size	Population	nsnp	build	PubMed ID
DNA methylation-estimated granulocyte proportions	34470	European	7289893	HG19/GRCh37	34187551
DNA methylation GrimAge acceleration	34467	European	7544493	HG19/GRCh37	34187551
DNA methylation Hannum age acceleration	34449	European	7541726	HG19/GRCh37	34187551
DNA methylation-estimated plasminogen activator inhibitor−1 levels	34448	European	7547661	HG19/GRCh37	34187551
DNA methylation PhenoAge acceleration	34463	European	7545555	HG19/GRCh37	34187551
DNA methylation GrimAge acceleration	34467	European	6196605	HG19/GRCh37	34187551
telomere length	472174	European	20134421	HG19/GRCh37	34611362
Intrinsic epigenetic age acceleration	34461	European	7544289	HG19/GRCh37	34187551
Aging	40229	European	-	-	31413261

### Outcome data

Sex-specific summary-level outcome data were obtained from a GWAS meta-analysis of IAs (cases with UIAs and aSAHs were also included), which contained 5140 cases and 71 934 controls from 23 cohorts of European ancestry. In addition, the effect of methylation-estimated phenotypes and telomere length on aging was also studied by employing aging as an outcome.

*To formally assess potential bias from sample overlap, we estimated the expected bias using the formula: bias ≈ (n_overlap / n_case) × (1 - specificity), as described by Burgess et al. Given the maximum potential overlap of < 4% and the high specificity of case definitions in the IA GWAS, the expected bias was negligible (<0.5% change in effect estimates) (* Burgess et al., 2016 *). This further supports that our findings are unlikely to be materially affected by sample overlap.*

### Statistical analysis

When the number of instrumental variants was more than three, multiple models were utilized to conduct MR analysis on the data, which includes the inverse variance weighted model (IVW), MR Egger model, and weighted median model. If instrumental variants were less than three, only the IVW model or the wald ratio method were used to assess the causal effects. As this technique assumed all instruments to be valid, some sensitivity analyses were carried out for testing the validity of the instrumental variants. In the inverse variance weighted model, the Cochran Q statistic was initially employed for evaluating the heterogeneity across variant-specific estimates.

*For exposures with fewer than three IVs (DNA methylation-estimated plasminogen activator inhibitor−1 levels, n = 2), only the Wald ratio or IVW method was used. F-statistics for all exposures exceeded 10, indicating no evidence of weak instrument bias.*

Then the horizontal pleiotropy was studied using the MR-Egger and MR-Pleiotropy Residual Sum and Outlier methods (Verbanck et al., 2018, Bowden et al., 2015). A significant nonzero Egger intercept indicates directional horizontal pleiotropy. With the assumption that the size of the pleiotropic effects is independent of the SNP-exposure effects (InSIDE-assumption), the bias was consequently tested and corrected due to directional pleiotropic impacts. The MR-Egger estimate may result in low statistical power when predicting a causal impact owing to its increased sensitivity to outliers and influential data points. After finding and removing the existing outlier SNPs, the effect estimate was calculated using MR-Pleiotropy Residual Sum and Outlier. An MR-Steiger direction and a weighted median-based MR analysis were also conducted, showing that more than half of the instrumental variants used in this research were valid.

Finally, all the causal effect estimates were converted to odds ratios (OR) with 95% CIs using the formulas below:

OR = EXP (beta)

95% CI = EXP (beta±1.96se)

When the results of the IVW method were significant, and either the MR egger or Weight median method was significant, a causal relationship exposure was observed with the outcome. All the analyses were conducted through R (3.6.2), using the R package Two Sample MR (0.5.6), Mendelian Randomization (0.5.1), and MRPRESSO (1.0). *An MR-Steiger test was conducted to verify the correct direction of causality for associations reaching nominal significance (P < 0.05). This test evaluates whether the variance in exposure explained by genetic instruments exceeds that explained in the outcome.*

## Results

### Relationship between DNA methylation-estimated phenotypes, telomere length, aging, and IAs

**Fig. 1:**
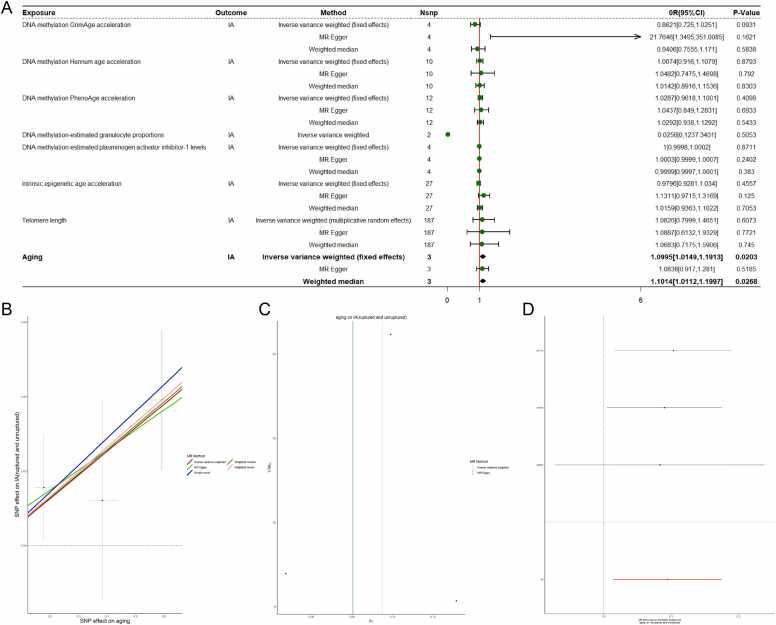
Effect estimates IA(ruptured and unruptured IAs). A, Investigation of the association of a genetically determined unit increase in exposure with the risk of IA using inverse-variance weighted, MR Egger, and weighted median estimates. B, Scatter plots of individual SNP effects and estimates from different MR techniques for the effect of aging on IA (ruptured and UIAs). C, Funnel plots of aging on IA (ruptured and UIAs). D, Leave-one-out analysis plots for aging on IA (ruptured and UIAs).

**Fig. 2:**
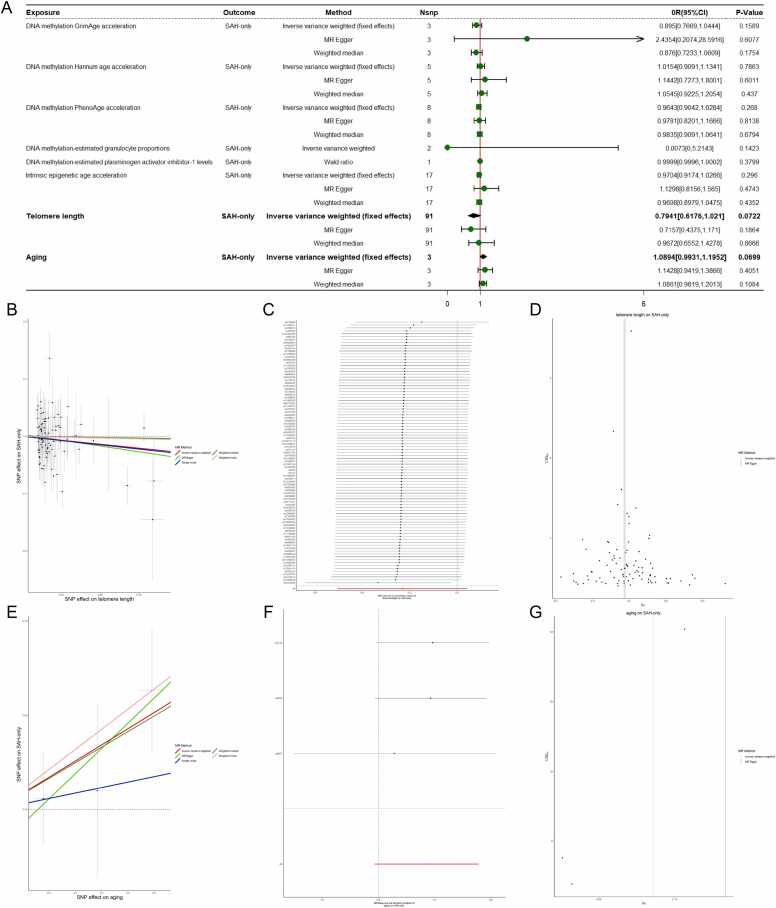
Effect estimates SAH-only. A, Investigation of the association of a genetically determined unit increase in exposure with the risk of SAH-only using inverse-variance weighted, MR Egger, and weighted median estimates. B, E, Scatter plots of independent SNP effects and various MR technique estimates representing the impact of telomere length on SAH-only (B) and aging on SAH-only (E). C, F, Leave-one-out analysis plots for telomere length on SAH-only (C) and aging on SAH-only (F). D, G, Funnel plots of telomere length on SAH-only (D) and aging on SAH-only (G).

The two-sample MR analysis showed a negative causal relationship between intrinsic epigenetic age acceleration and UIA, which means the increase of intrinsic epigenetic age acceleration could reduce the risk of UIAs (*P* = 0.022272, OR=0.9059[95%CI,0.83–0.99]). No causal relationship was found between other methylation-estimated phenotypes, telomere length, and IAs (*P* > 0.05). Suggestive evidence of a 9.95% increase in the risk of IAs was observed per 1-SD increase in aging (*P* = 0.020289, OR=1.099528[95%CI,1.01–1.19]), but this association doesn’t exist in UIA and SAH (Fig. 1, Fig. 2).

*After applying Bonferroni correction for multiple comparisons (8 exposures × 3 outcomes=24 tests; corrected α=0.05/24 =0.00208), the associations between IEAA and UIA (P = 0.022) and aging and aSAH (P = 0.020) did not reach statistical significance. These findings should therefore be interpreted as suggestive evidence requiring replication in independent cohorts.*

**Fig. 3:**
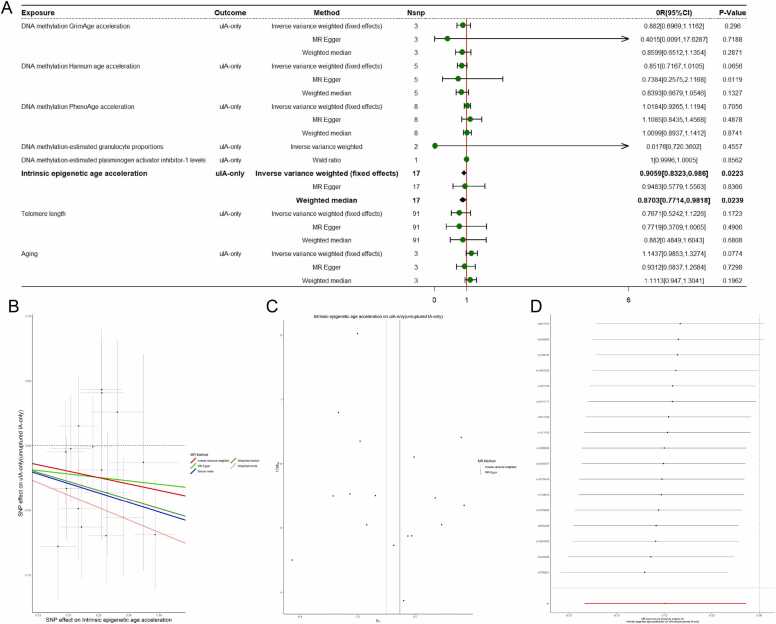
Effect estimates for Intrinsic epigenetic age acceleration (IEAA) on unruptured intracranial aneurysm (UIA-only). A, Forest plot showing causal effect estimates from three MR methods: inverse-variance weighted (IVW), MR-Egger, and weighted median. Each square represents the point estimate, with error bars indicating 95% confidence intervals. B, Scatter plot of individual SNP effects on IEAA (x-axis) versus UIA (y-axis). Each point represents one instrumental SNP. Slopes of the lines represent causal estimates from different MR methods: IVW (blue), MR-Egger (green), and weighted median (orange). C, Funnel plot for the IVW analysis. Asymmetry would suggest potential pleiotropy; approximate symmetry is observed here. D, Leave-one-out sensitivity analysis showing the effect estimate (IVW) after sequentially removing each SNP. No single SNP drives the overall estimate, supporting robustness.

*An MR-Steiger directionality test was performed to confirm the correct causal direction. For the IEAA-UIA association, the Steiger test indicated that the direction of causality was appropriate (Steiger P = 0.031), supporting the interpretation that IEAA influences UIA risk rather than the reverse (* Fig. 3 *).*

### Relationship between DNA methylation-estimated phenotypes, telomere length, and aging

**Fig. 4:**
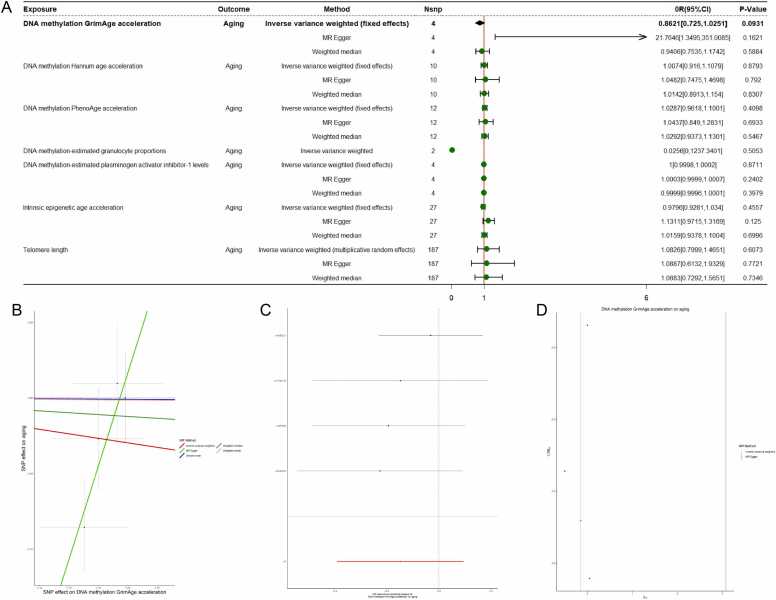
Effect estimates Aging. A, Investigation of the association of a genetically determined unit increase in exposure with the risk of aging using inverse-variance weighted, MR Egger, and weighted median estimates. B, Scatter plots of independent SNP effects and various MR technique estimates representing the impact of DNA methylation GrimAge acceleration on aging. C, Leave-one-out analysis plots for DNA methylation GrimAge acceleration on aging. D, Funnel plots of DNA methylation GrimAge acceleration on aging.

**Fig. 5:**
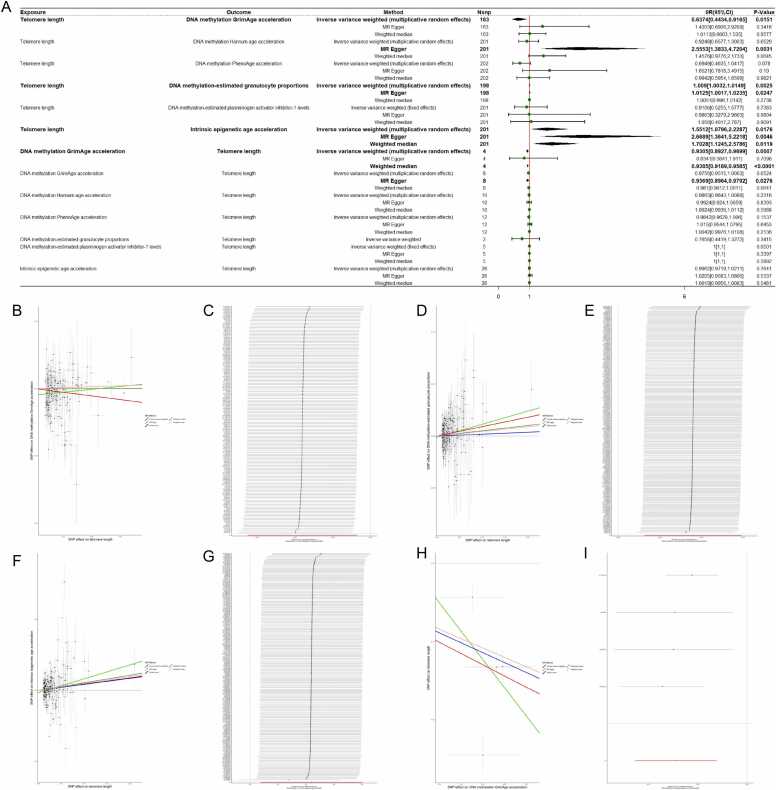
Effect estimates DNA methylation-estimated phenotypes (DNA methylation-estimated granulocyte proportions, DNA methylation GrimAge acceleration, DNA methylation Hannum age acceleration, DNA methylation-estimated plasminogen activator inhibitor−1 levels, DNA methylation PhenoAge acceleration, and Intrinsic epigenetic age acceleration) and telomere length. A, Investigation of the association of a genetically determined unit rise in exposure with the risk of DNA methylation-estimated phenotypes and telomere length using inverse-variance weighted, MR Egger, and weighted median estimations. B, Scatter plots of independent SNP effects and various MR technique estimates representing the impact of telomere length on DNA methylation GrimAge acceleration. C, Leave-one-out analysis plots for telomere length on DNA methylation GrimAge acceleration. D, Scatter plots of independent SNP effects and various MR technique estimates representing the impact of telomere length on DNA methylation-estimated granulocyte proportions. E, Leave-one-out analysis plots for telomere length on DNA methylation-estimated granulocyte proportions. F, Scatter plots of independent SNP effects and various MR technique estimates representing the impact of telomere length on DNA methylation GrimAge acceleration. G, Leave-one-out analysis plots for telomere length on DNA methylation GrimAge acceleration. H, Scatter plots of independent SNP effects and various MR technique estimates representing the impact of DNA methylation GrimAge acceleration on telomere length. I, Leave-one-out analysis plots for DNA methylation GrimAge acceleration on telomere length.

To further clarify the link between DNA methylation-estimated phenotype, telomere length, and longevity, a two-sample MR analysis was carried out using methylation-estimated phenotypes and telomere length as exposure and aging as an outcome. The findings showed no remarkable causal relationship between methylation-estimated phenotype and telomere length and aging. Multiple associations exist between telomere length and DNA methylation-estimated phenotype. However, a positive causal relationship was found between telomere length and DNA methylation-estimated granulocyte proportions and intrinsic epigenetic age acceleration. This means that each 1-SD increase in telomere length resulted in a 0.9% increase in the DNA methylation-estimated granulocyte proportions (*P* = 0.002518, OR=1.009041 [1.003166,1.01495]) and a 55% increase in the intrinsic epigenetic age acceleration (*P* = 0.017597, OR=1.55152 [1.079562,1.551152]). In contrast, increased DNA methylation GrimAge acceleration decreases telomere length (*P* = 0.00066, OR=0.930501 [0.892715,0.969886]) (Fig. 4, Fig. 5).

## Discussion

With GWAS, thousands of genetic loci linked to various complex traits, diseases, and illnesses have been found (Dong et al., 2021). The majority of the alterations in the genome can be recorded cost-effectively by genotyping only several hundred thousand variants and afterward equating non-genotyped variants using a compact genotyping reference panel owing to the GWAS paradigm, which utilizes the linkage disequilibrium (LD) correlation structure of the genome (Pavlides et al., 2016). Nevertheless, due to the LD structure, the correlations frequently point to genomic regions with many genes, making it exceedingly challenging to prioritize these genes to find the most functionally relevant ones using GWAS data alone. Given the possible causative variations in a typical genome-wide substantial locus, laboratory-based tracking of associated areas is expensive and prohibitive.

For the first time, a Mendelian randomization method based on DNA methylation-estimated phenotypes, telomere length, and aging pooled data was applied to a pooled IA dataset during this research to identify their pleiotropic or causality-induced traits and associations with IA risk. This research aimed to investigate whether DNA methylation-estimated phenotypes, telomere length, and aging were associated with IAs (including aneurysmal SAH versus unruptured aneurysms). Six epigenetic age indices were considered, namely DNA methylation-estimated phenotypes: DNA methylation-estimated granulocyte proportions, DNA methylation GrimAge acceleration, DNA methylation Hannum age acceleration, DNA methylation-estimated plasminogen activator inhibitor−1 levels, DNA methylation PhenoAge acceleration and Intrinsic epigenetic age acceleration.

A negative causal relationship between intrinsic apparent age acceleration (IEAA) and UIAs suggested that increased intrinsic apparent age acceleration decreases the risk of UIAs. However, no other epigenetic clock estimation variable, telomere length, was significant with UIA. The IEAA clock is a derivative of the Horvath pan-tissue predictor that regresses DNA methylation-based estimates for naïve and depleted CD8+T cells, plasma cells, CD4+T cells, natural killer cells, monocytes, and granulocytes. While other cell subtypes may affect the outcomes of this research, Changes in cell composition are not likely to impact GWAS outcomes shared among IEAA and other epigenetic clocks (Hannum clock, PhenoAge, and GrimAge) (McCartney et al., 2021).

Nevertheless, GWAS outcomes for IEAA and GrimAge remained enriched with previous leukocyte GWAS findings, including basophils. Mendelian randomization analysis showed putative causality of lymphocyte counts for PhenoAge, GrimAge, and Hannum age acceleration but not for IEAA. It was shown that IEAA and PhenoAge acceleration had established roles in several aging/regenerative phenotypes (McCartney et al., 2021, Gontier et al., 2018). Viola Vaccarino et al. showed that depression is associated with higher levels of DNAm age acceleration (Liu et al., 2022). It has also been shown that women with ischemic stroke have a lower biological age acceleration than males (Gallego-Fabrega et al., 2022).

In addition, aging has a causal relationship with the risk of IA, i.e., increasing age also causes an increased risk of disease, which emphasizes the potential role of aging in IAs. However, there was no significant causal relationship between UIAs and SAH. Interestingly, James Feghali et al. found a mean age of 52.8 years (standard deviation = 15.0 years) in a cohort of 1671 patients with ruptured aneurysms. The proportion of patients aged 80 years or older increased approximately four folds (*P* < 0.001) over a 7-year progressive interval over 28 years, with the mean patient age increasing from 51.2 to 54.6 years (*P* = 0.002). Analysis of patients with aSAH showed an increase in patient age over time, with a significant increase in the proportion of patients in their eighties and a decrease in the proportion of patients under 50 years of age (Feghali et al., 2021).

Moreover, a two-sample Mendelian randomization analysis was conducted by employing aging as the endpoint to further clarify the relationship between DNA methylation-estimated phenotypes, telomere length, and aging. There were multiple associations between telomere length and DNA methylation-estimated phenotypes, with a positive causal relationship between changes in telomere length and DNA methylation-estimated granulocyte proportions and intrinsic epigenetic age acceleration. This may imply that a rise in telomere length can result in an elevation in DNA methylation-estimated granulocyte proportions and intrinsic epigenetic age acceleration. In turn, an increase in DNA methylation GrimAge acceleration can lead to a decrease in telomere length.

Many epigenetic aging indices have been established previously, shifting away from those validated largely based on actual age (such as the Horvath epigenetic clock) and toward those verified or optimized for disease occurrence and biological risk factors. GrimAgeAccel is calculated by regressing the residuals of GrimAge on actual age. GrimAge is developed as a composite marker based on 7 plasma proteins (adrenomedullin, beta−2-microglobulin, cystatin-C, growth differentiation factor 15, leptin, fibrinogen activator inhibitor 1 and tissue inhibitor metalloproteinase 1) and smoking pack years, sex and epigenetic proxy markers (Ross et al., 2020), exhibiting a strong relationship with death.

dnam PAI−1, dnam ADM, and dnam cystatin C are epigenetic substitution markers for fibrinogen activator inhibitor 1, adrenomedullin, and cystatin-C, respectively (Ross et al., 2020). Fibrinogen activator inhibitor−1 is a glycoprotein that inhibits fibrinolysis or thrombolysis, which can promote IA formation (Labeyrie et al., 2017). It has also been shown that the fibrinogen activator inhibitor−1 4 G allele in the 4 G/5 G promoter polymorphism enhances cerebral ischemia following aneurysmal SAH (Vergouwen et al., 2004). Therefore, in clinical practice, treatment aimed at enhancing fibrinolysis after early aneurysm occlusion may effectively prevent and treat cerebral ischemia in individuals suffering from aneurysmal SAH. Adrenomedullin is a vasodilator peptide hormone that may play a regulatory role in cerebral vasospasm and subsequent cerebral ischemia after SAH (Fujioka et al., 2000).

A protein called cystatin-C is commonly employed as a measure of renal function and is a valuable predictor of IA development and rupture (Maimaiti et al., 2022). Another study showed that cystatin C expression decreased with the progression of the aneurysm (Turhon et al., 2022). The moderate association among DNAm PAI−1, DNAm ADM, and DNAm cystatin C and their corresponding plasma biomarkers in the study by Ross, K. M et al. suggests that these indices may capture additional biological activity (Ross et al., 2020). Therefore, additional robust studies are needed before definitive conclusions can be devised. Future research should focus on determining the specific biological activity or process that the epigenetic age acceleration index captures and how it might relate to IAs.

*To our knowledge, this is the first study to assess the association* of epigenetic age acceleration index, telomere length, and aging with IA outcomes. Notably, this research adds to the emerging research area, suggesting that increased intrinsic epigenetic age acceleration decreases the risk of developing UIAs. Therefore, further analyses can emphasize identifying key epigenetic molecular markers of intrinsic epigenetic age acceleration that could influence the probability of UIAs by modulating their expression. MR investigations are ahead of conventional observational research in reducing the possibility of residual confounding. Furthermore, this study offered novel perspectives that might aid in understanding how gender disparities function in IA prevalence (UIA and aSAH). In addition, numerous sensitivity analyses were carried out using recently available large-scale GWAS data to evaluate the robustness of the research's findings.

*It should be noted that after correction for multiple testing, the observed associations did not remain statistically significant. Thus, these findings are presented as suggestive rather than definitive, and replication in larger, independent datasets is warranted before drawing firm conclusions.*

*The finding that increased IEAA may reduce the risk of UIAs is counterintuitive, as epigenetic age acceleration has typically been associated with adverse health outcomes, including cardiovascular disease, cognitive decline, and mortality. Several potential explanations warrant consideration. First, this finding may reflect cell-type composition artifacts, as IEAA is derived from algorithms that adjust for measured immune cell proportions; however, unmeasured cell subtypes could influence the results. Second, it is possible that certain aspects of biological aging, such as cellular senescence, might exert protective effects in the vascular wall by limiting proliferative capacity and reducing the formation of aneurysmal lesions, although this hypothesis remains speculative. Third, residual confounding or reverse causality cannot be entirely excluded, despite the MR-Steiger test supporting the direction of causality. Given that this association did not remain significant after multiple testing correction, it should be interpreted as suggestive rather than definitive. Future studies using larger sample sizes and alternative epigenetic clock measures are needed to validate this finding.*

The MR design of this study has the benefit of being less susceptible to bias than conventional observational studies. Other significant benefits are the comparatively high number of intracranial aneurysm cases and the enhanced adaptability of the study's findings to include polymorphic variants in several MR sensitivity analyses. Five MR methods and heterogeneity tests were included to avoid potential pleiotropic effects. Population stratification bias was minimized as all analyses were restricted to populations of European ancestry and adjusted for in the original genome-wide association study using pooled data.

There are several limitations to this research. First, data on all relevant exposure SNPs were unavailable in the resulting GWAS despite searching for possible surrogates. Therefore, many exposure SNPs could not be employed in this MR study. Secondly, there is a lack of studying how DNA methylation-estimated phenotypes, telomere length, and aging function in the pathogenesis of IAs from the transcriptome level.

Finally, the alterations used for epigenetic clocks such as IEEA and GrimAge reflect their lifelong effects on IAs, whereas, in contrast, clinical interventions typically exert greater changes in IAs. Caution should be exercised when inferring the effects of clinical interventions from MR, and clinical trials are required to determine optimal practices. This MR study proves that (i) increased intrinsic apparent age acceleration decreases the risk of developing UIAs. (ii) There is a causal association between aging and IA risk, i.e., as age increases, so does disease risk. (iii) Increased telomere length leads to an increase in the proportion of granulocytes estimated by DNA methylation and an inherent acceleration in epigenetic age. (iv) An accelerated increase in DNA methylation GrimAge leads to decreased telomere length. These findings add to the existing evidence and highlight the causal role of DNA methylation-estimated phenotypes, telomere length, aging, and IA occurrence.

## Conclusion

In conclusion, as of current knowledge, this MR research is the first to assess the association of DNA methylation-estimated phenotypes, telomere length, and aging with IAs. An increase in intrinsic apparent age acceleration decreases the risk of UIAs, whereas other epigenetic clock estimation variables (GrimAge acceleration, Hannum age acceleration, PhenoAge acceleration, estimated granulocyte proportions, and estimated plasminogen activator inhibitor−1 levels) and telomere length were not associated with UIA.

## Abbreviations

IA: intracranial aneurysm; UIA: unruptured intracranial aneurysm; SAH: subarachnoid hemorrhage; GWAS: Genome-wide association; MR: Mendelian Randomization; SHBG: sex hormone-binding globulin; S-Ca: serum calcium; S−25OHD: 25-hydroxyvitamin D; CpG: cytosine-phosphor-guanine; DNAm age: DNA methylation age; TL: Telomere length; IVW:Inverse variance weighted model; OR: odds ratios; LD: linkage disequilibrium; IEAA: Intrinsic epigenetic age acceleration.

## CRediT authorship contribution statement

**Aierpati Maimaiti:** Writing – review & editing, Writing – original draft, Data curation, Conceptualization. **Xin Zhang:** Writing – review & editing, Writing – original draft, Visualization, Validation, Project administration, Methodology, Investigation, Formal analysis, Data curation, Conceptualization. **Mirzat Turhon:** Data curation, Conceptualization. **Meixia Xie:** Data curation, Conceptualization. **Wenbao Liang:** Writing – review & editing, Writing – original draft, Software, Resources, Project administration, Methodology, Investigation, Funding acquisition, Data curation, Conceptualization. **Naibijiang Yalikun:** Writing – original draft, Visualization, Data curation, Conceptualization.

## Informed consent and ethical approval

This MR study was based on public summary data from published GWAS that had already obtained informed consent and ethical review board approvals. This study was completed in accordance with the Helsinki Declaration as revised in 2013.

## Consent for publication

Not applicable.

## Trial registration

Not applicable.

## Funding

This work was supported by the Xinjiang Key Laboratory of Neurological Disorder Research (Grant No. XJDX1711-2428) and the Natural Science Foundation of Xinjiang Uygur Autonomous Region (General Program, Grant No. 2024D01C119).

## Conflicts of Interest

We do not have any competing financial interests or personal relationships.

## Data Availability

The article/Supplementary material contains the original contributions made to the study. Corresponding authors can be contacted for more information.

## Appendix A Supplementary material

Supplementary Figure 1 An overall design of the present study

## Footnotes

Appendix A Supplementary data associated with this article can be found in the online version at doi:10.1016/j.ibneur.2026.05.009.
