# Supplementary figures and images for: DNA methylation-estimated phenotypes, telomere length, aging and risk of intracranial aneurysms: Evidence from genetic studies
Source: IBRO Neurosci Rep. 2026 May 30;21:1–10. doi: 10.1016/j.ibneur.2026.05.009 (PMC13251712; doi:10.1016/j.ibneur.2026.05.009)

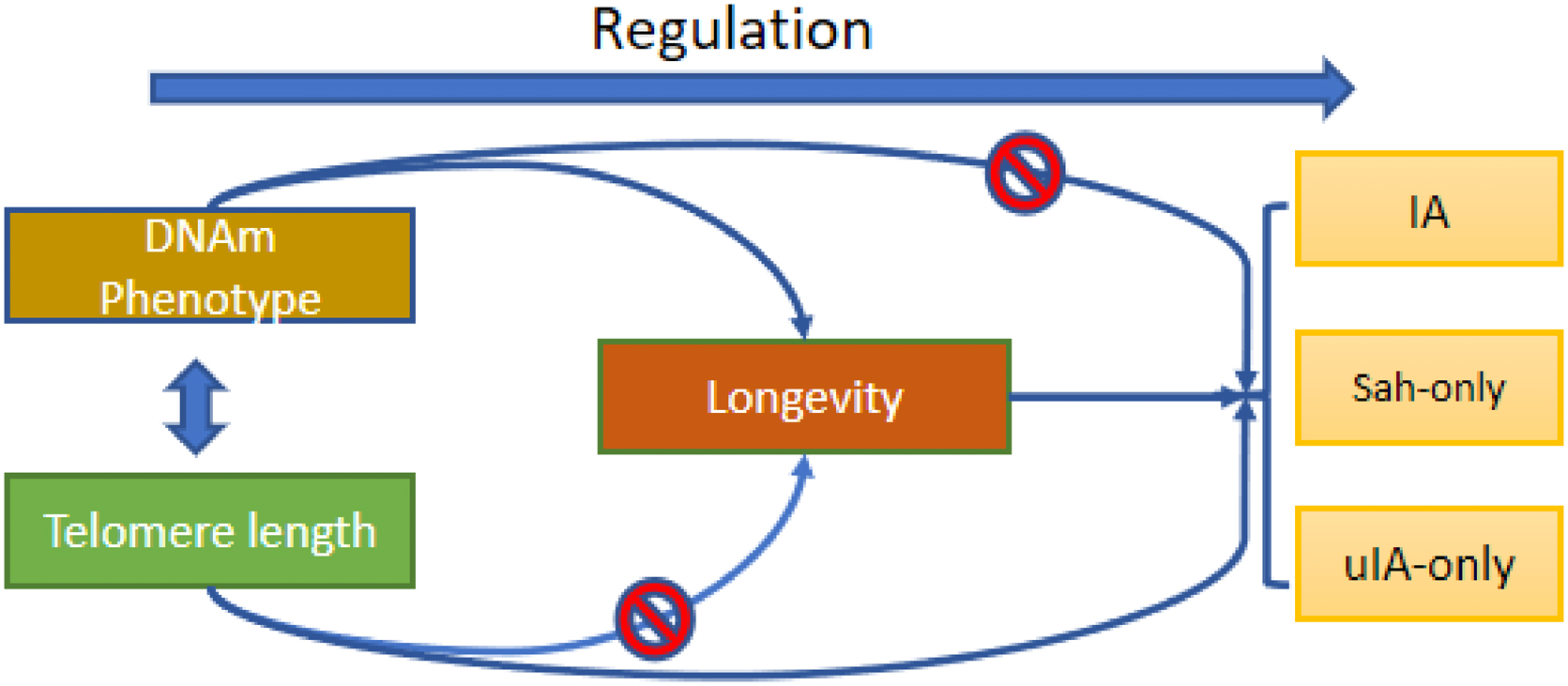

Supplement: Supplementary Figure 1 — An overall design of the present study [file mmc1.jpg]
